# Medical Service in the British Army and Navy

**Published:** 1859-08

**Authors:** 


*Medical Service in the British Army and Navy.*—Inequality of rank with other officers, and insufficient pay, has long been a subject of complaint in the medical service of the British army and navy. By the following we see that the government has made an important, and, it is generally thought, a wise change in this respect.

*Rank and Pay of the Medical Officers in the British Navy.*—The following warrant has lately been issued for the future regulation of the rank, pay, &c., of the medical officers of the British Navy:—

"At Buckingham Palace, May 13. Present—The Queen's Most Excellent Majesty in Council.

"Whereas there was this day read at the Board a memorial from the Right Honorable the Lords Commissioners of the Admiralty, dated the 12th of May, 1859, in the words following, viz.: Whereas, we have had under our consideration the necessity of assimilating, as far as possible, the relative ranks and rates of pay and half pay of the medical officers of your Majesty's navy and army: And whereas, we are of opinion that it would be for the advantage of your Majesty's service that the following regulations should be adopted for the medical officers of the Royal Navy, viz:—

"1st. That there shall be four grades of medical officers, viz.: 1. Inspector-general of hospitals and fleets. 2. Deputy inspector-general of hospitals and fleets. 3. Surgeon, who, after twenty years' service on full pay, ten of which in the rank of surgeon, shall be styled Staff Surgeon. 4. Assistant Surgeon.

2d. That no candidate shall be admitted to the examination for a commission in the medical department of the Royal Navy who does not possess such a diploma as would qualify a civilian to practice medicine and surgery; and no such candidate shall receive a commission as assistant surgeon until he shall have satisfactorily passed an examination in naval surgery and hygiene before a board of examiners appointed by the Lords Commissioners of the Admiralty.

"3d. That no assistant surgeon shall be eligible for promotion to the rank of surgeon until he shall have passed such examination as the Lords Commissioners of the Admiralty may require, and shall have served on full pay with the commission of assistant surgeon for five years, of which two, at least, shall have been passed on board one of your Majesty's sea-going ships.

"4th. That no surgeon shall be eligible for promotion to the rank of deputy inspector-general of hospitals and fleets until he shall have served ten years in your Majesty's navy on full pay, of which three, at least, must have been passed in one of your Majesty's ships on some one or more foreign stations, with the rank of surgeon.

"5th. That no deputy inspector-general of hospitals and fleets shall be eligible for promotion to the rank of inspector-general until he shall have served five years at home, or three years abroad, in the rank of deputy inspector-general. That in cases of emergency, however, or when the good of your Majesty's service may render such alteration desirable, it shall be competent for the Lords Commissioners of the Admiralty to shorten the several periods of service above-mentioned, in such manner as they shall deem fit and expedient.

"6th. That the rates of full pay of the medical officers of your Majesty's navy shall, in future, be in accordance with the following schedule:—

FULL PAY.

| RANK. | After 80 years' service on full pay. | After 25 years' service on full pay. | After 20 years' service on full pay. | After 15 years' service on full pay. | After 10 years' service on full pay. | After 5 years' service on full pay. | Under 5 years' service on full pay. |
|---|---|---|---|---|---|---|---|
| | £ s. d. | £ s. d. | £ s. d. | £ s. d. | £ s. d. | £ s. d. | £ s. d. |
| Inspector-General of Hospitals and Fleets . . . . . . | 2 5 0 | 2 5 0 | 2 0 0* | | | | |
| Deputy Inspector-General of Hospitals and Fleets . . . . | 1 14 0 | 1 10 0 | 1 8 0* | | | | |
| Staff-Surgeon . . . . . | .. | 1 5 0 | 1 2 0 | | | | |
| Surgeon . . . . . . . | .. | .. | .. | 0 18 0 | 0 15 0* | | |
| Assistant-Surgeon . . . . | .. | .. | .. | .. | 0 13 0 | 0 11 6 | 0 10 0 |

"7th. That every medical officer on the active list, now on half pay and those who may be placed on it subsequently to the date of your Majesty's Order in Council authorizing this proposal, shall be allowed the half pay to which his period of service on full pay shall entitle him, according to the following schedule:—

* Or on promotion, should these periods of service not have been already completed.

HALF PAY.

| RANK. | After 30 years' service on full pay. | After 25 years' service on full pay. | After 20 years' service on full pay. | After 15 years' service on full pay. | After 10 years' service on full pay. | After 5 years' service on full pay. | Under 5 years' service on full pay. |
|---|---|---|---|---|---|---|---|
| | £ s. d. | £ s. d. | £ s. d. | £ s. d. | £ s. d. | £ s. d. | £ s. d. |
| Inspector-General of Hospitals and Fleets . . . . . . . | 1 17 6 | 1 13 6 | 1 10 0* | | | | |
| Deputy Inspector-General of Hospitals and Fleets . . . . . | 1 5 6 | 1 2 6 | 1 1 0* | | | | |
| Staff-Surgeon . . . . . . | .. | 0 18 6 | 0 16 6 | | | | |
| Surgeon . . . . . . . | .. | .. | .. | 0 13 6 | 0 11 0* | | |
| Assistant-Surgeon . . . . . | .. | .. | .. | .. | 0 11 0 | 0 8 0 | 0 6 0 |

"8th. That with a view to maintain the efficiency of the service, all medical officers of the ranks of staff surgeon, surgeon, and assistant surgeon, shall be placed on the retired list when they shall have attained the age of sixty years; deputy inspectors-general shall be placed on such retired list when they shall have attained the age of sixty-five years; and inspectors-general when they shall have attained the age of seventy years. Officers thus superannuated shall receive the rates of half pay mentioned in the preceding schedule.

"9th. That the relative rank of the medical officers of your Majesty's navy shall be similar to that conferred upon the medical officers of the army, and shall be as follows:—

"An assistant surgeon shall rank as a lieutenant in the army, according to the date of his commission; and after six years' service on full pay, as a captain in the army, according to the date of the completion of such service.

"A surgeon shall rank as major in the army, according to the date of his commission; and a staff surgeon as lieutenant-colonel, but junior of that rank.

"A deputy inspector-general of hospitals and fleets shall rank as lieutenant-colonel, according to the date of his commission; and after five years' service on full pay as deputy inspector-general, shall rank as colonel, according to the date of completion of such service.

"An inspector-general of hospitals and fleets shall rank as brigadier-general, according to the date of his commission; and after three years' service on full pay, as inspector-general, shall rank as major-general, according to the date of completion of such service.

"Provided always that no naval medical officer, while borne on the books of one of her Majesty's ships, or employed on establishments on shore, shall

* Or on promotion, should these periods of service not have been already completed.

be deemed superior in rank to the officer appointed to command such ship or establishment; but such commanding officer shall, under all circumstances, be held to be superior in rank and precedence to every officer under his command.

"10th. That such relative rank shall carry with it all precedence and advantages attaching to the rank with which it corresponds, and shall regulate the choice of quarters, rates of lodging-money, servants, forage, fuel and light, or allowances in their stead, when medical officers of the navy may be employed on shore on joint service with your Majesty's land forces; but that medical officers serving in the fleet shall, notwithstanding the relative rank thus conferred upon them, in all such details, and also in matters relating to the duties of the fleet, and the discipline and interior economy of your Majesty's ships, be subject, as heretofore, to the authority of any executive officer of the military branch while on duty, under the general regulations which may from time to time be prescribed by the Lord High Admiral, or the commissioners for executing the office of Lord High Admiral; and that medical officers shall share prize money according to the proclamation or proclamations which may be in force for the time being, for regulating the distribution of the proceeds of prizes in the Royal Navy.

"11th. That medical officers shall be entitled to the same allowances, on account of wounds and injuries received in action, as combatant officers holding the same relative ranks.

"12th. That the families of medical officers shall in like manner be entitled to the same allowances as granted to the families of combatant officers holding the same relative ranks.

"13th. That medical officers shall be held entitled to the same honors as other officers of the Royal Navy of equal relative rank.*

"14th. That a medical officer retiring after a full pay service of twenty-five years, may, in cases of distinguished service, receive a step of honorary rank, but without increase of half pay.

"15th. That good service pensions shall be awarded to the most meritorious medical officers of the Royal Navy, under such regulations as shall from time to time be determined upon, on the recommendation of the Lord High Admiral, or the commissioners for executing that office.

"16th. That four of the most meritorious medical officers of the Royal Navy shall be named honorary physicians, and four honorary surgeons, to your Majesty.

---

* This clause does not extend to the compliments to be paid by garrison or regimental guards, as laid down in pages 29 and 30 of your Majesty's regulations for the army, nor to corresponding honors paid on board your Majesty's ships.

"We do, therefore, most humbly submit that your Majesty will be graciously pleased, by your Order in Council, to grant us the necessary authority for carrying the foregoing regulations into effect; the Lords Commissioners of your Majesty's Treasury having signified their concurrence."

Her Majesty having taken the said memorial into consideration, was pleased, by and with the advice of her Privy Council, to approve of what is therein proposed; and the Right Honorable the Lords Commissioners of the Admiralty are to give the necessary directions herein accordingly. WM. L. BATHURST.—*Med. Times and Gaz.*, June 4, 1859.

